# An Ulcerated Large Colic Lipoma Causing Rectorrhagia Treated With Endoscopic Loop-Assisted Resection

**DOI:** 10.7759/cureus.14321

**Published:** 2021-04-06

**Authors:** Maddalena Zippi, Antonella Toma, Nada Paoluzi, Francesca Maccioni, Roberta Pica

**Affiliations:** 1 Unit of Gastroenterology and Digestive Endoscopy, Sandro Pertini Hospital, Rome, ITA; 2 Unit of Urgent Digestive Endoscopy, Sandro Pertini Hospital, Rome, ITA; 3 Department of Radiological Sciences, Oncology and Pathology, Sapienza University, Policlinico Umberto I, Rome, ITA

**Keywords:** colic lipoma, en-bloc resection, endoloop, endoscopic resection, rectorrhagia

## Abstract

Colonic lipomatous polyps are often an incidental finding during colonoscopy. Generally, these types of polyps can cause gastrointestinal bleeding when they are larger than 4 cm in size. Some case reports have documented the occurrence of overlying adenomatous formations in the apical portion, as well as ulcerated mucosa. There is currently no standardized endoscopic removal technique for their treatment. In this report, we present a case of a large and ulcerated lipoma causing rectorrhagia, which was successfully treated with endoscopic en-bloc resection and endoloop placement.

## Introduction

Colonic lipomas, which are recognized as non-epithelial benign tumors of mesenchymal origin, generally represent an incidental finding during colonoscopy. Their reported incidence varies from 0.2-4.4% [1]. Although most of them are asymptomatic, they can become clinically manifest in 6% of cases [2]. In particular, polyps that are bigger than 2 cm can cause alterations in bowel habits or abdominal pain, whereas those that are bigger than 4 cm may lead to colorectal intussusception or obstruction or gastrointestinal bleeding due to ulceration of the same and episodes of intussusception [2,3,4]. In the past, the resective treatment of these lesions was purely surgical in nature. Nowadays, with the emergence of new techniques and devices, endoscopy has assumed a pivotal role in their treatment, enabling less invasive and easily repeatable complex procedures. In this report, we describe a case of a man who came to our attention due to rectal bleeding; his colonoscopy showed the presence of a large and ulcerated lipoma in the cecum, which was treated endoscopically with loop-assisted resection.

## Case presentation

**Figure 1:**
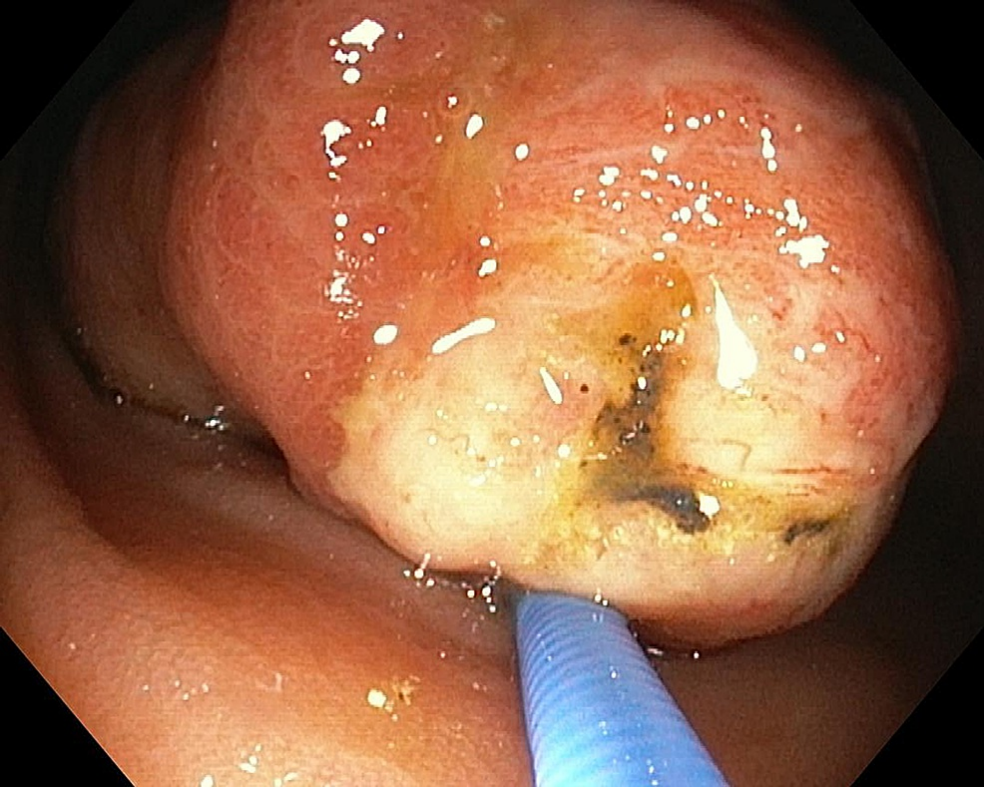
Endoscopic view: head of the polyp raised by biopsy forceps

A 74-year-old male was admitted due to rectorrhagia. The patient had been undergoing regular therapy with an antiplatelet agent (clopidogrel 75 mg/day). On admission, his temperature was 37.2 °C, the blood pressure was 115/75 mmHg, the pulse rate was 85/minute, and his respiratory rate was 16/minute. Laboratory exams were remarkable for the presence of anemia only (hemoglobin level of 11.2 g/dl). Physical examination excluded signs of peritoneal reaction to palpation. At that point, it was decided to perform an urgent colonoscopy, which revealed both fresh and coagulated blood along all the colonic tracts and, in addition to the presence of diverticular orifices in the sigma, a large pedunculated submucosal lipoma of about 5 cm in the cecum, whose apex, after repeated washing, presented hematin and fibrin, related to ulcerated mucosa (Figure 1).

**Figure 2:**
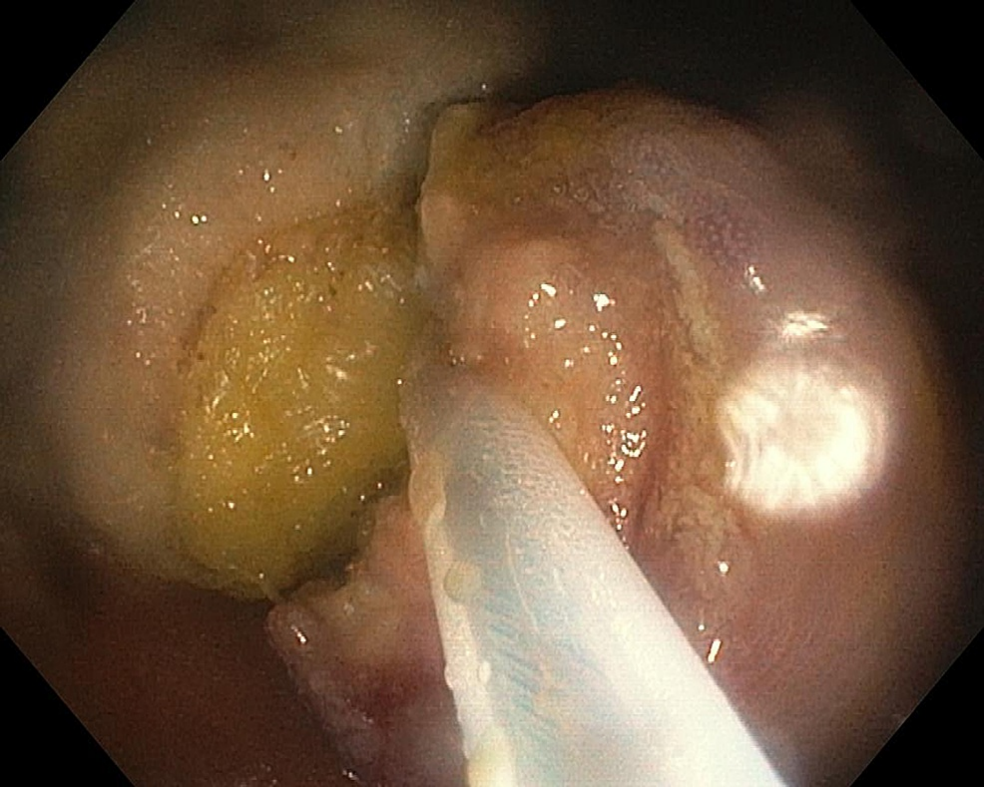
Endoscopic view: mucosal en-bloc resection using polypectomy snare The typical lipomatous component with the characteristic yellow color can be seen

**Figure 3:**
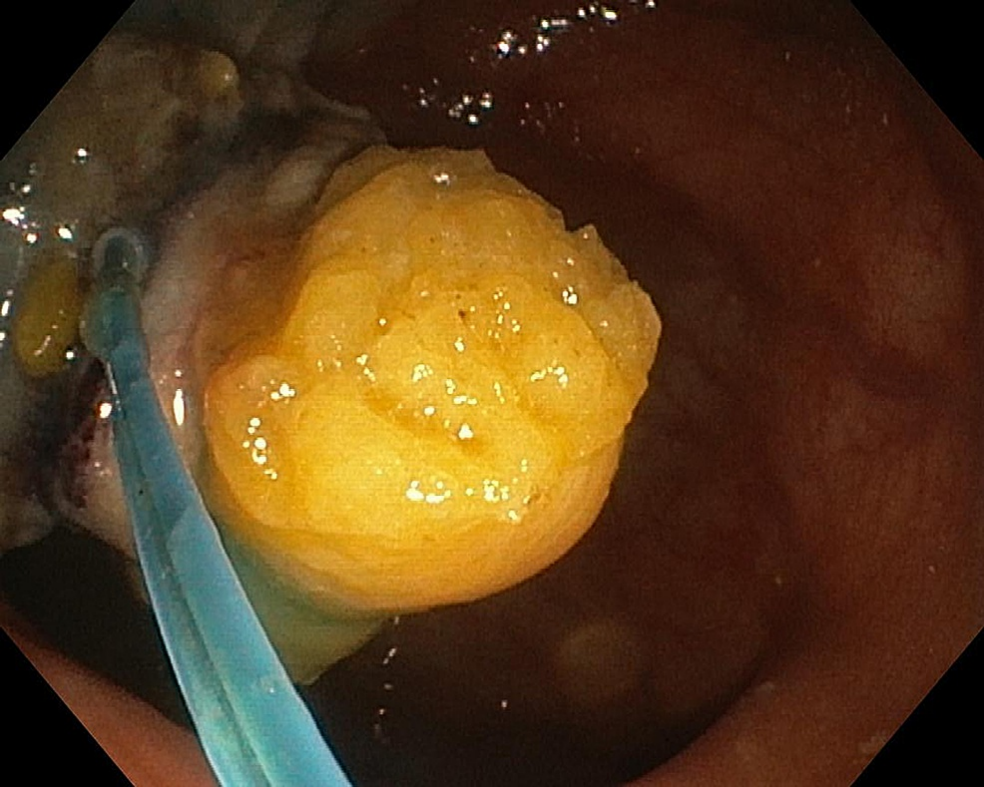
Endoscopic view: ligation of the lipoma’s base with a nylon snare

A second endoscopy, after a five-day suspension of the antiplatelet agent, was performed under sedation with propofol and also with the use of carbon dioxide. Informed consent was obtained prior to the procedure. The apex of the polypoid formation was removed with endoscopic mucosal en-bloc resection (AcuSnare® Polypectomy Snare, AS-1S, 2.5 x 5.5 cm, Cook Medical, Bloomington, IN), using the setting of forced coag of 60 watts (Endo-cut Q, Erbe Elektromedizin GmbH, Tübingen, Germany) (Figure 2), after both infiltration of the mucosae, at the base of the lipomatous polyp with saline-diluted adrenaline (1:10,000) mix methylene blue, and the placement of a detachable endoloop (single-use ligating device, HX-400U-30, 30mm®, Olympus Medical System Corp., Tokyo, Japan) (Figure 3).

**Figure 4:**
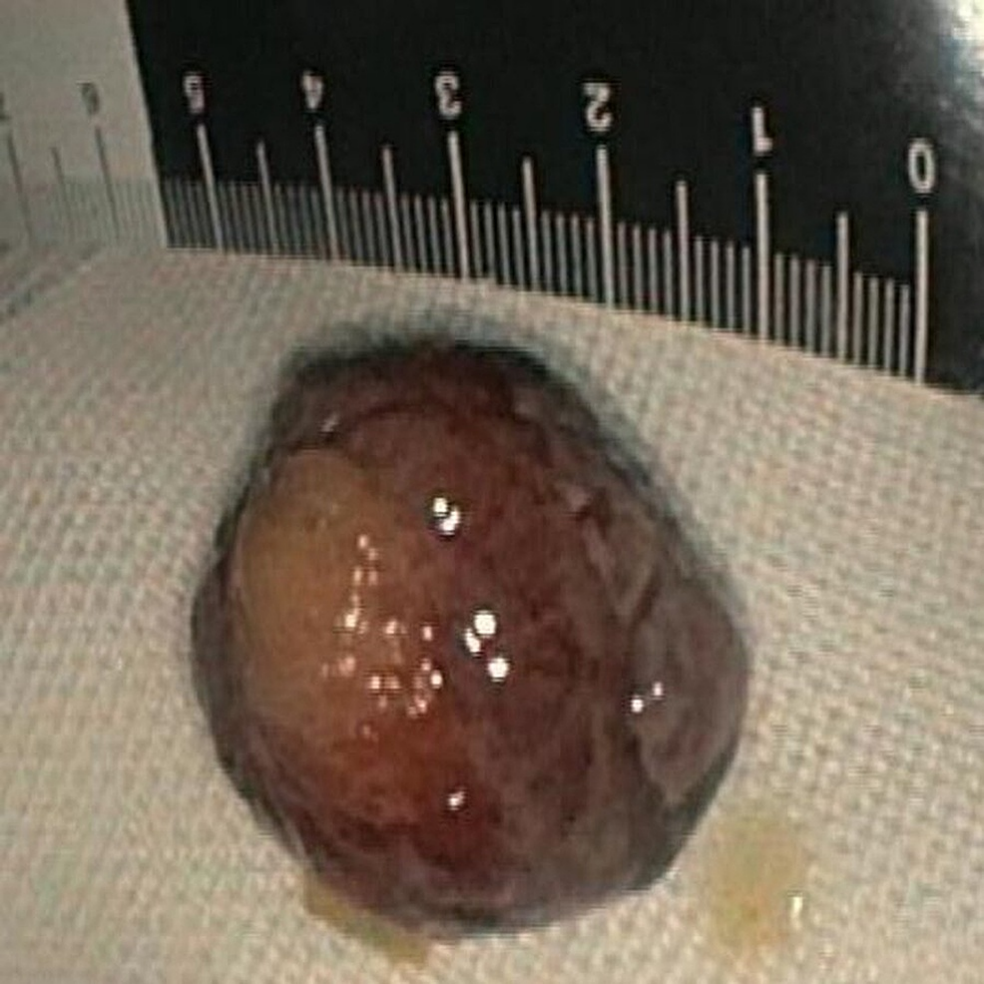
Resected lipoma after electrocoagulation It measured 30 mm in size

At the end of the procedure, the retrieved specimen measured 3 cm (Figure 4). The related histopathological findings showed, beyond the typical adipose tissue proliferation, signs of an ulcerated mucosa without the presence of dysplasia and adenomatous or serrated components. Broad-spectrum antibiotics were administered after the procedure. The patient responded well to the treatment and was soon asymptomatic with no bleeding and/or perforation, and he was discharged two days after in good condition.

## Discussion

Colon lipomas are non-epithelial submucosal tumors and are predominantly located in the right colon and cecum in approximately 90% of the cases [5]. Endoscopically, they appear as polypoid formations with a smooth, slightly yellow, rounded appearance, with broad-based attachment, covered with normal or eroded mucosa. Colon lipomas originate from the connective tissue of the intestinal walls and most commonly arise from the submucosa layer [5]. These rare polypoid-like formations are asymptomatic in 75% of patients, but when major symptoms or lesions occur, these need to be removed [6]. Surgical resection is preferred in the case of large lipomas with a broad implant base or in cases where the histological examination of the biopsy has not been decisive concerning a malignant lesion [7].

In a systematic review of 88 articles regarding symptomatic large lipomas, 127 patients were analyzed in light of their clinical course [8]. As a clinical presentation, rectal bleeding was present in 46 cases (46%). Fifty-nine patients (46%) underwent endoscopic resection procedure, while 68 (53%) underwent elective surgery: open surgery for 52 patients (76%) and laparoscopic procedure for 16 (23%) [8]. The pertinent question here is as follows: which is the best approach to endoscopically resect colonic lipomatous polyps that are larger than 4 cm in size? Some studies have shown that endoscopic resection of lipomas that are >2 cm in diameter is associated with a greater risk of perforation due to the adipose tissue, which is an inefficient conductor for electric current and extends the cutting time [9]. Several techniques have been proposed, comprising unroofing, dissection-based techniques, endoscopic mucosal resection (EMR), and loop-assisted-snare resection. In the last approach, the prophylactic use of an endoloop may prevent post-polypectomy bleeding [10]. In a recent systematic review of 24 studies, a total of 77 lipomatous formations were removed using the following techniques: EMR (40.3%), loop-assisted-snare resection (37.7%), unroofing (13%), and dissection-based technique (9.1%) [11]. No statistical significance was observed with regard to the clinical remission rates among patients in all groups. Patients who underwent loop-assisted techniques developed adverse events in 13.8% of cases [11].

## Conclusions

This case highlights how endoloop could be used with excellent results to prevent both early and late bleeding related to endoscopic resection of large colic lipoma by expert endoscopists. Therapeutic digestive endoscopy has the advantage of being an easily repeatable procedure compared to surgery, and it allows for the treatment of most post-procedural complications quickly.
